# Does Rodent Management Impact the Distribution of *Rattus rattus* and *Mastomys natalensis* in Village Settings?

**DOI:** 10.1111/1749-4877.70015

**Published:** 2025-11-11

**Authors:** Herieth Mkomwa, Rhodes Makundi, Steven R. Belmain, Aurore Ponchon, Mwajabu Selemani, Alfan A. Rija, Marcela P.A. Espinaze, Sandra Telfer

**Affiliations:** 1 Department of Wildlife Management, Sokoine University of Agriculture, Morogoro, Tanzania; 2 African Centre of Excellence for Innovative Rodent Pest Management and Biosensor Technology Development, Sokoine University of Agriculture, Morogoro, Tanzania; 3 Institute of Pest Management, Sokoine University of Agriculture, Morogoro, Tanzania Building, University of Aberdeen, Aberdeen, UK; 4 Natural Resources Institute, University of Greenwich, Kent, UK; 5 School of Biological Sciences, Zoology Building, University of Aberdeen, Aberdeen, UK

**Keywords:** abundance, intensive trapping, movement, rodent control

## Abstract

Rodents are significant agricultural and public health pests in rural village settings. Traditional rodent control methods, such as poison baiting and locally made traps, are usually implemented by individual households when rodent abundance is high and often provide only short-term relief. Moreover, impacts on different pest species may vary, and changes to inter-specific interactions may have potential ecological consequences. This study examines the impact of community-wide daily trapping inside houses on the abundance and distribution of *Rattus rattus* and *Mastomys natalensis* in Kilombero District, Tanzania. In this area, *R. rattus* are typically found inside houses, while *M. natalensis* are found outside. Snap traps were deployed daily inside all houses in treatment villages, while control villages received no intervention. Rhodamine B (RhB) baits in exterior areas monitored the movements of rodents from these areas to houses. Intensive in-house trapping successfully reduced the abundance of *R. rattus* but not *M. natalensis. M. natalensis* increased its use of houses in treatment villages, with a significant rise in the proportion of individuals captured indoors and a significant increase in the proportion of these individuals that had consumed RhB compared to non-treatment villages. Our results suggest that *M. natalensis* benefits from the reduced presence of *R. rattus* by expanding its habitat use to include houses. These findings underscore the effectiveness of intensive trapping in controlling *R. rattus* but reveal potential ecological readjustments following community ecology principles, with habitat shifts by *M. natalensis*. This highlights the need for integrated, multi-species management approaches for sustainable rodent pest control.

## 1 Introduction

Rodents are notorious pests worldwide, causing significant damage to agriculture, stored food, and infrastructure (Makundi et al. 1999; Stenseth et al. 2003; Gebresilassie et al. 2006; Singleton et al. 2010; Swanepoel et al. 2017). Their adaptability allows them to inhabit a variety of environments, ranging from crop fields to residential areas (Ralaizafisoloarivony et al. 2014; Ssuuna et al. 2020). In many low- and middle-income countries, rodents pose a serious threat to food security. Estimates suggest that rodents are responsible for up to 20%–30% loss in grain storage and up to 5%–15% damage in field crops annually (Makundi et al. 1999; Singleton et al. 2010). These losses significantly reduce income for smallholder farmers and threaten the overall sustainability of agricultural systems. In addition to their agricultural and economic impacts, rodents are known vectors of various zoonotic diseases, creating substantial public health challenges (Meerburg et al. 2009). Given their wide economic and health impacts, particularly in rural villages, the need for effective rodent control becomes critical.

Over the years, various control methods have been implemented to mitigate the negative impacts of rodent species. Traditional control measures, including keeping cats as natural predators, the use of poison baits with locally available commercial rodenticide mixed with food, locally made pitfall traps, and environmental sanitation, are the most practiced approaches (Makundi and Massawe 2011; Smith et al. 2015). However, these strategies often provide only short-term relief (Makundi et al. 1999). The need for more sustainable and ecologically sound rodent management methods has led to the adoption of ecologically based rodent management (EBRM) strategies and integrated pest management (IPM) approaches. EBRM interventions were notably introduced in African countries such as Tanzania, Swaziland, and Namibia to provide local solutions. Intervention actions, including community-based intensive trapping of rodents, use of trap barrier systems in agricultural fields, habitat manipulation, and sanitary measures, have demonstrated that the impacts of rodents on communities could be drastically reduced (Makundi and Massawe 2011; Singleton et al. 2004). Unlike toxic chemical control methods, EBRM emphasizes long-term prevention and ecosystem health, making it a more effective and sustainable solution for rodent management (Singleton et al. 2005; Brown et al. 2006; Jacob et al. 2007). For instance, a study by Makundi et al. (1999) demonstrated that ecological knowledge combined with management strategies led to more sustainable rodent population reductions. Similarly, Singleton et al. (2004) found that implementing trapping and habitat modification methods in ways informed by an understanding of rodent movement patterns and breeding cycles resulted in more effective rodent control in agricultural systems.

In most cases, when management is conducted, including EBRM, control is usually only possible in a spatially restricted area. Compensatory immigration from surrounding areas can therefore lead to rapid recovery of populations. Moreover, in many areas, multiple rodent pest species may exist. Control in spatially restricted areas may therefore have differential impacts on different species, potentially resulting in compensatory dynamics, with less impacted species shifting behavior to exploit the vacant ecological niche (Brown et al. 2001). This can exacerbate the damage to agricultural systems and complicate control efforts. Similarly, intra-specific variation in behavior and habitat use may result in spatially restricted management removing a biased subset of pest populations. These issues highlight the complexity of rodent population management and underscore the need for integrated, long-term strategies.

Among the most widespread and problematic rodent species in sub-Saharan Africa are black rats *Rattus rattus* and multimammate rats *Mastomys natalensis*. Although they are sympatric, these two species exhibit distinct behaviors and habitat preferences that influence their management. *R. rattus* typically inhabits human dwellings and peri-domestic environments, making them frequent pests within homes, where they contaminate food and damage structures (Gebresilassie et al. 2006; Massawe et al. 2011; Monadjem et al. 2011; Awoniyi et al. 2021). In contrast, *M. natalensis* predominantly thrives in agricultural fields, causing extensive damage to crops such as maize, rice, and cassava (Makundi and Massawe 2011). These habitat preferences have typically been identified through trapping studies. Recently, a study used Rhodamine B (RhB) baits to explore movements of the two species within village contexts (Mkomwa et al. 2024). RhB is a non-toxic dye that can be observed on the nose and mouthparts using an ultraviolet torch (Fry et al. 2009). The findings suggested that both species may actually frequently move between their preferred and non-preferred habitats (Mkomwa et al. 2024). This cross-habitat connectivity complicates effective management strategies in village settings, as it may trigger potential compensatory dynamics, where species from non-target habitats may repopulate targeted areas.

Here, we examine how community-wide daily trapping inside houses affects the distribution, abundance, and movement of *R. rattus* and *M. natalensis* in rural villages of Kilombero district, Tanzania. Given that trapping studies indicate houses are the preferred habitat of *R. rattus*, while *M. natalensis* are caught more frequently in exterior habitats, our first hypothesis was that this management approach would be more effective against *R. rattus* than *M. natalensis*, with the treatment impacting the abundance of *R. rattus* inside houses but also in the surrounding habitats due to the houses acting as a source population. Our second hypothesis was that removing predominantly *R. rattus* individuals from inside houses could impact the behavior of *M. natalensis*, increasing the use of houses by *M. natalensis*. Our third hypothesis was that, if there were intra-specific differences in habitat use, the daily trapping would be expected to preferentially remove the individuals of each species that use the houses to a greater extent. In this case, removing those individuals could decrease the proportion of individuals caught in this habitat for both species. The findings will contribute to the development of more sustainable, long-term rodent management practices that can improve food security and public health in rural villages.

## 2 Materials and Methods

### 2.1 Study Area

This study was conducted in Kilombero District, Morogoro Region, Tanzania, covering an area of 14 918 km^2^. Kilombero is located between latitudes 7°40′S and 9°21′S and longitudes 35°20′E and 37°48′E, with an average elevation of 208m above sea level. The district’s geography includes a vast floodplain, supporting agriculture and livestock farming, with rice and sugarcane being the predominant crops. The climate is characterized by distinct wet and dry seasons, with four sub-seasons: the hot wet season (December to March), the cool wet season (April to June), the cool dry season (July to August), and the hot dry season (September to November). Annual rainfall ranges between 1200 and 1800 mm, and temperatures vary from 26°C to 32°C.

**Figure 1:**
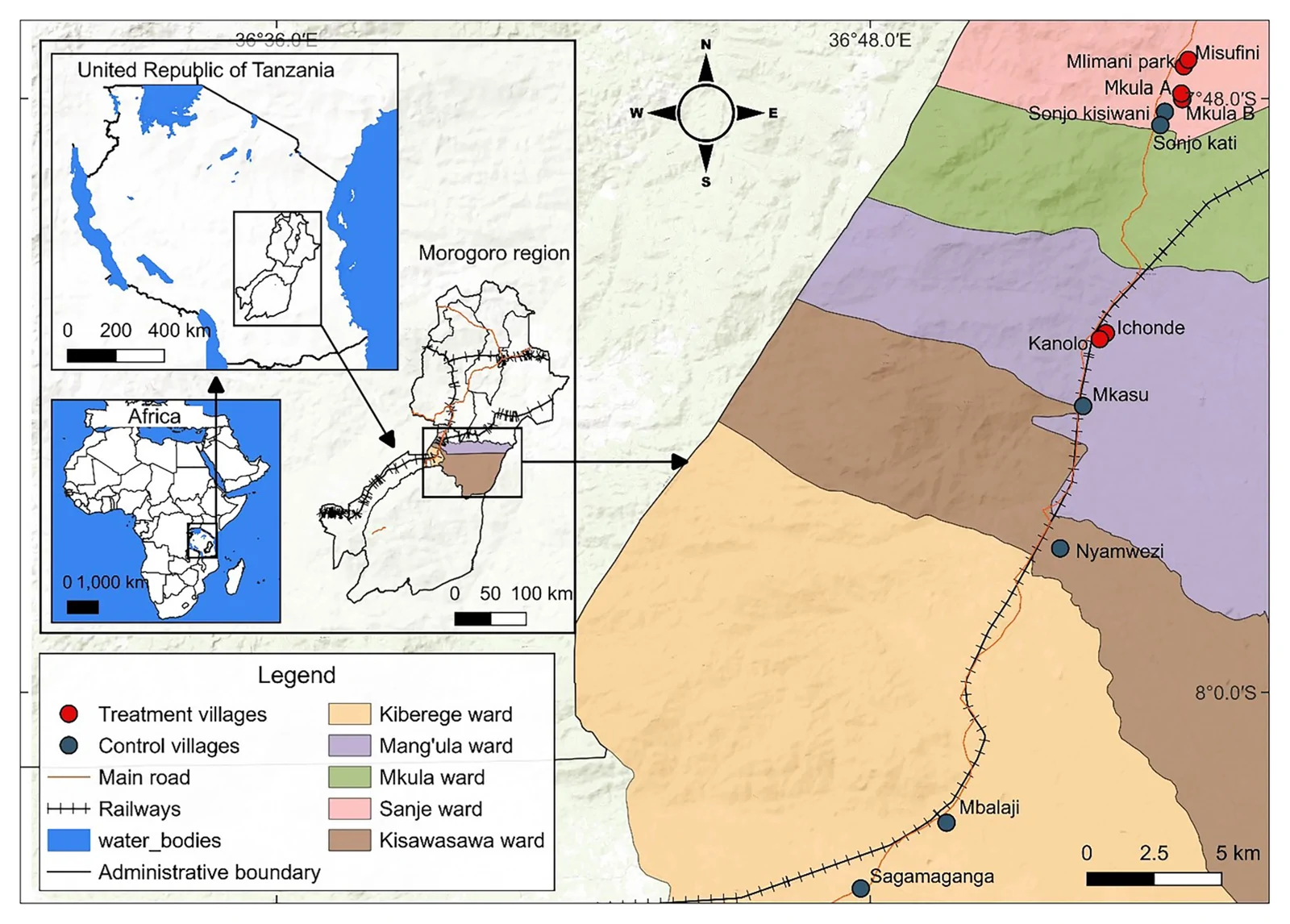
Map of Tanzania showing study areas in Kilombero District, Morogoro Region.

Twelve villages were selected for this study, including Mkula A, Mkula B, Kanolo, Misufini, Ichonde, Mlimani Park, Sonjo Kisiwani, Mbalaji, Nyamwezi, Mkasu, Sagamaganga, and Sonjo Kati (Figure 1). The study was conducted from May 2022 to November 2023.

### 2.2 Community-Wide Trapping Intervention

The intervention consisted of a community-based intensive trapping intervention. Each household in the treatment villages received two snap traps, which were placed indoors and checked daily by household members. They were encouraged to reset the traps each morning. Daily follow-up visits by trained field assistants (members from the community) facilitated implementation and supported troubleshooting. Prior to the intervention, sensitization meetings were conducted to explain the objectives of the study, and basic training sessions were provided to demonstrate how to set snap traps safely and effectively.

Initially, from May 2022, the intervention was conducted in three villages (Mkula A, Mkula B, and Kanolo), with no intensive community-implemented trapping conducted in the other villages (non-treatment villages). In April 2023, the intervention was expanded to include Ichonde, and in June 2023, the intervention was expanded to Mlimani Park and Misufini. The remaining six villages continued to serve as non-treatment villages. In non-treatment villages, households were able to practice their normal uncoordinated rodent control methods.

### 2.3 Monitoring Trapping and RhB Baits in Villages

To evaluate the effect of treatment on the distribution, relative abundance, and movement of rodents within and around all the villages, we conducted monitoring trapping sessions, combined with the use of RhB baits. These trapping sessions were conducted by the research team in June 2022, August 2022, November 2022, February 2023, May 2023, August 2023, and November 2023. Full details of the trapping design are given in Mkomwa et al. (2024), which reports a wider study to explore movement patterns of *M. natalensis* and *R. rattus* within and around villages using RhB baits. Briefly, the trapping focused on three distinct habitat types: inside houses, peridomestic areas (<10 m from the house), and crop fields/vegetated areas close to the village (within 70 m of houses). These reflected a gradient across all study villages, from the interior of houses to peridomestic areas and crop fields. Peridomestic areas included the immediate surroundings of homes where small household activities occurred (e.g., cooking, storage, and gardening), while crop fields extended further out and comprised both cultivated and uncultivated vegetated areas. Trapping points outside were located close to shelter (e.g., vegetation), and the minimum distance between trapping points was 10 m. At each trapping point, we used one Sherman (Tallahassee, FL, USA) and one modified box trap (locally made single capture live trap), apart from June and August 2022, when inside house trapping points only had modified box traps. Some of the villages had RhB bait placed outside houses (Mkula A, Mkula B, Kanolo, Misufini, Mlimani Park, and Ichonde), while the rest of the villages had RhB bait placed inside houses. RhB baits of approximately 20 g (see Mkomwa et al. 2024 for details) were placed daily for 2–3 days prior to trapping sessions, with signs of consumption checked each morning. For villages with RhB bait placed outside, bait stations were located close to water taps, with up to five water taps selected evenly across the village. Villages with RhB bait placed inside houses had three RhB baits placed inside each of the 10 households distributed throughout the village. Monitoring trapping started 1 day after the removal of the RhB bait. For villages that had RhB bait placed outside houses, trapping was conducted at 18 trapping points per RhB station as follows: 4 within houses, 4 in peridomestic areas, and 10 in crop fields/vegetation areas. For villages with RhB bait placed inside houses, trapping was conducted at nine trapping points per RhB station as follows: one within the house, four in the peri-domestic areas, and four in crop fields/vegetated areas. For the first seasonal trapping, from June 2022 to August 2022, traps were set for three to six nights, whereas from November 2022 to November 2023, traps were only set for four consecutive nights.

### 2.4 Collection of Data on Captured Animals

Traps were inspected every morning. Upon capture, rodents were euthanized humanely using halothane in accordance with ethical guidelines. Animals were sacrificed to allow the collection of organs for a related study on rodent-borne disease, and because releasing pest rodents within houses and village environments is not feasible. Species identification followed the keys by Happold (2013). Each animal was examined for evidence of RhB consumption using an ultraviolet torch (Fry et al. 2009). Observation was done in the field, using hands to provide shade and enhance the visibility of fluorescence. RhB fluorescence was frequently observed in the nasal, mouth, and eye areas, and we assume that any fluorescence in these body parts of a captured animal was evidence of consumption of the bait within the same field session in the last 2–8 days.

### 2.5 Statistical Analyses

As some villages only conducted the community-implemented daily trapping within houses in the second year of the study, but not in the first year, we created a binary variable for “treatment” denoting whether intensive daily trapping had taken place inside houses within the village during the last 30 days or not. To test our first hypothesis, we investigate the effect of treatment on the abundance of *R. rattus* and *M. natalensis*, using trap success during monitoring trapping sessions as an indicator of relative abundance for each rodent species in each habitat type (inside houses, peridomestic areas, and areas of external vegetation or crop fields). Trap success was defined as the number of individuals of the species captured per 100 trap nights.

### 2.6 Trap Success Rate = (Number of Captured Individuals/Number of Trap Nights) × 100

*Kruskal–Wallis tests* were used to evaluate the effect of treatment on the relative abundance of each species within each habitat. We hypothesized that intensive daily trapping within houses would have a greater effect on *R. rattus* than *M. natalensis*, as the widespread distribution and high numbers of *M. natalensis* in exterior habitats were likely to allow replacement of any individuals removed, while in contrast, the removal of *R. rattus* from their preferred habitat inside houses would reduce the number of individuals emigrating into the surrounding habitats.

To explore our second and third hypotheses, we assess the effect of treatment on the distribution and movement of *R. rattus* and *M. natalensis* across the different habitats. We used Chi-square tests or, where counts were low, Fisher tests, based on the raw counts of animals caught in each habitat. For each species, we examined whether the proportion of individuals captured in each habitat differed between treatment and non-treatment villages. For each species and each of the habitat types, we also examined whether there were significant differences in RhB consumption rates between treatment and non-treatment villages (with analyses restricted to villages with RhB bait placed outside). Under our second hypothesis, that *M. natalensis* from outside areas would use houses more following the removal of *R. rattus*, we expected in treatment villages an increase in the proportion of *M. natalensis* being caught inside during our monitoring trapping, and an increase in the proportion of *M. natalensis* caught inside houses that had consumed RhB bait (compared to non-treatment villages). Our third hypothesis related to intraspecific differences in habitat use. If there were *R. rattus* individuals preferentially using outside habitats rather than inside habitats, we expected that these animals would be less likely to be trapped during the daily intensive trapping, and in this case, the proportion of *R. rattus* caught outside during our monitoring trapping and the proportion of *R. rattus* that had consumed RhB bait could increase with treatment. Conversely, if there were *M. natalensis* individuals preferentially using inside habitats, rather than the outside habitats typically utilized by this species, we expected these individuals to be removed, and therefore, if these individuals were not replaced by others (as expected under our second hypothesis), the proportion of *M. natalensis* caught inside could decrease with treatment.

All statistical analyses were conducted using R version 4.2.3 (R Core Team 2023).

## 3 Results

### 3.1 Effect of Treatment on Relative Abundance

**Figure 2:**
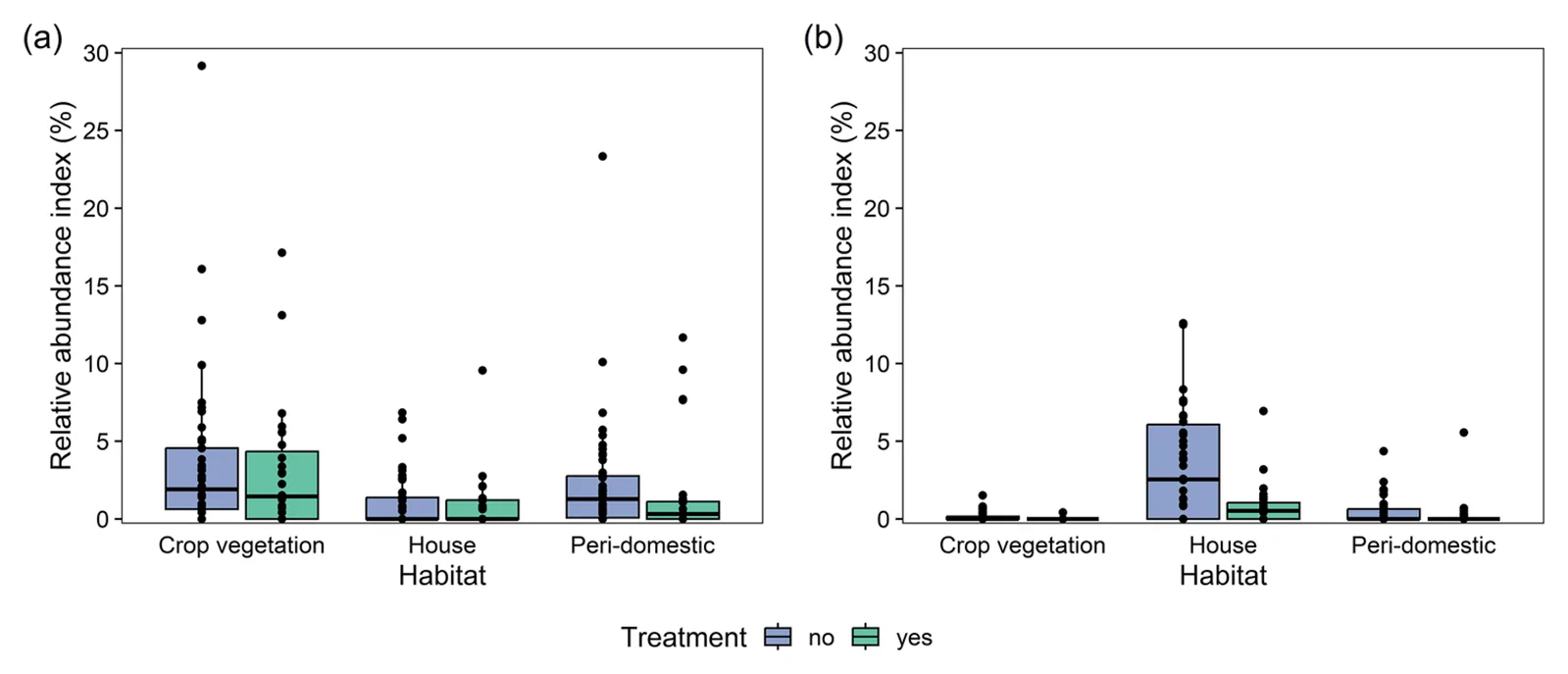
Boxplots of *Mastomys natalensis* (a) and *Rattus rattus* (b) within each habitat of the treatment (yes) and non-treatment villages (no). Yes = villages that have been conducting community-wide daily trapping inside houses during the 30 days prior to the monitoring trapping session. The box represents the interquartile range, with the line inside the box representing the median.

There was a significant reduction in the relative abundance of *R. rattus* in treatment villages compared to non-treatment villages inside houses (*H* = 10.38, df = 1, *p* < 0.01). As expected under our first hypothesis, this effect of treatment extended to the surrounding habitats, with a significant reduction in the relative abundance of *R. rattus* in crop fields (*H* = 3.95, df = 1, *p* = 0.04). Although there was also a suggestion of a decline in peri-domestic areas (Figure 2b), this was not statistically significant (*H* = 2.12, df = 1, *p* = 0.14). In contrast, as expected, we found no evidence of a reduction in the relative abundance of *M. natalensis* even inside houses (Figure 2a; inside houses *H* = 0.20, df = 1, *p* = 0.66).

### 3.2 Effect of Treatment on Distribution and Movement Between Habitats

**Figure 3:**
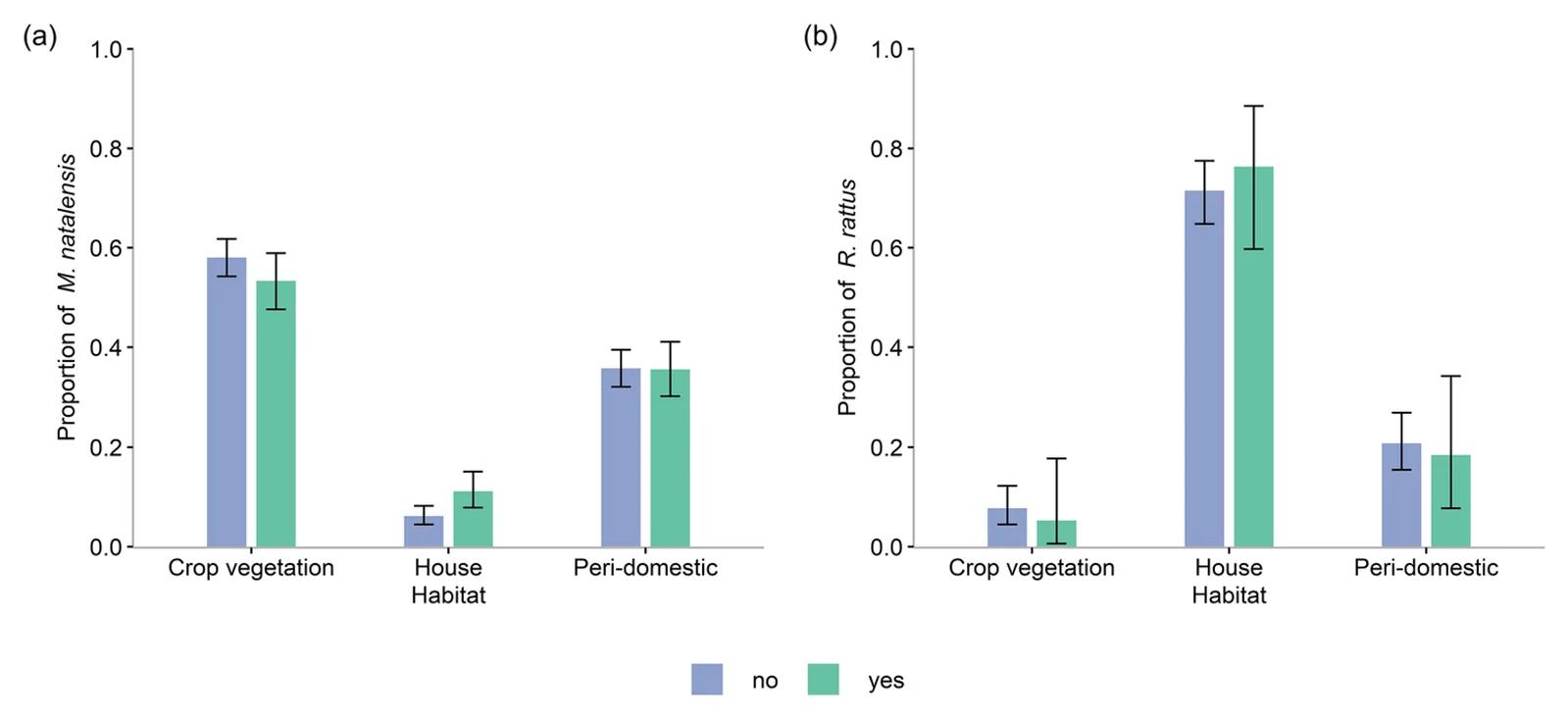
Mean proportion ± 95% CI of *Mastomys natalensis* (a) and *Rattus rattus* (b) caught in each habitat, split by treatment. Yes = villages that have been conducting community-wide daily trapping inside houses during the 30 days prior to the monitoring trapping session.

**Figure 4:**
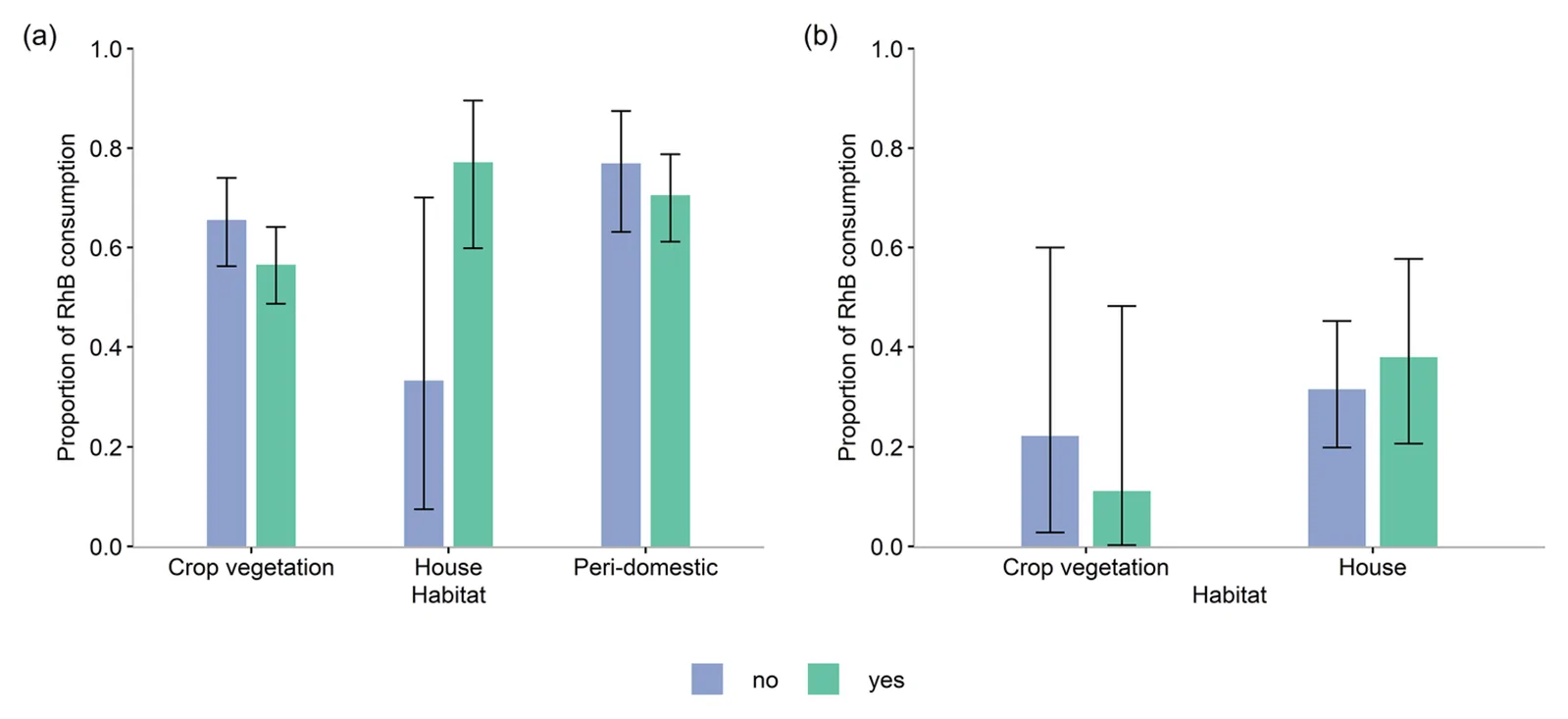
Mean proportion ± 95% CI of *Mastomys natalensis* (a) and *Rattus rattus* (b), which had consumed RhB bait, split by habitat and treatment. Due to the small proportion of *R. rattus* captured in peridomestic and crop field/vegetated areas (Figure 2), these groups were combined. Yes = villages that have been conducting community-wide daily trapping inside houses during the 30 days prior to the monitoring trapping session.

Supporting our second hypothesis, the treatment did appear to increase the use of houses by *M. natalensis*, with a significant increase in the proportion of *M. natalensis* captured inside houses in villages that had conducted daily trapping within houses during the last 30 days compared to villages that had not (Figure 3a; *χ*^2^ = 7.70, df = 2, *p* = 0.02), and a significant increase in the proportion of *M. natalensis* captured inside houses that had evidence of RhB bait consumption (Figure 4a; Fishers exact test, *p* = 0.02). There was no evidence that the proportion of *M. natalensis* captured in peridomestic or crop vegetation habitats that had consumed RhB bait differed between treatment and non-treatment villages (peridomestic: *χ*^2^ = 0.44, df = 1, *p* = 0.51; crop vegetation: *χ*^2^ = 2.00, df = 1, *p* = 0.16). For *R. rattus*, in contrast, we did not observe any change in the distribution of rats across the habitat types (Figure 3b; Fishers exact test, *p* = 0.86), and the proportion of animals that had consumed RhB bait did not differ between treatment and non-treatment villages in any of the habitat types (houses: *χ*^2^ = 0.12, df = 1, *p* = 0.73; crop vegetation: Fishers exact test, *p* = 0.45) (Figure 4b). Based on these results, we found no evidence supporting our third hypothesis that intraspecific differences in habitat use and removal of individuals using the houses could result in an increase in the proportion of *R. rattus* that were caught outside and a decrease in the proportion of *M. natalensis* that were caught inside.

## 4 Discussion

Our study provides new insights into the impacts of localized trapping inside houses on the relative abundance, distribution, and movement of *R. rattus* and *M. natalensis* in rural village settings in eastern Africa. Consistent with our hypothesis, intensive trapping within houses significantly reduced *R. rattus* abundance, particularly in residential areas. Such reductions are consistent with those from previous studies, indicating that targeted localized trapping is effective in reducing rodent populations within preferred habitats (Rahelinirina et al. 2021; Eisen et al. 2018). The reduction in the population of *R. rattus* indoors also led to their decline in abundance in crop fields, and there was no increase in the proportion of *R. rattus* caught outside in villages that had been conducting the treatment. These findings do not support the hypothesis of individual specialization in habitat use within *R. rattus*, instead suggesting that the reduction in indoor *R. rattus* leads to fewer cross-habitat incursions. This highlights that targeting a species in its preferred habitat can have impacts on surrounding areas or habitats.

Daily intensive trapping was less effective for *M. natalensis*, which is more commonly found in exterior habitats. The relative abundance of *M. natalensis* did not decrease in response to inhouse trapping, likely due to compensatory immigration from untreated nearby habitats, where *M. natalensis* thrives. Our findings are consistent with our expectation that targeting a species such as *M. natalensis* in its non-preferred habitat is less effective at reducing abundance. However, an increased proportion of *M. natalensis* was caught inside houses in treatment villages, which may suggest a potential shift in habitat use by this species in response to reduced *R. rattus* competition. This apparent redistribution of *M. natalensis* toward residential areas may indicate an interspecific response, whereby reductions in *R. rattus* presence influence *M. natalensis* habitat preferences, as has been observed in other ecosystems where small subordinate species persist with larger dominant competitors (Chock et al. 2022). This shift in habitat use by *M. natalensis* suggests that reductions in *R. rattus* populations may indirectly open up residential areas as exploitable environments for other rodent species, supporting previous observations on interspecies competition and habitat overlap (Cordero et al. 2024; Granjon et al. 2023). Furthermore, a significant proportion of *M. natalensis* captured inside houses had evidence of consuming RhB bait, which further emphasizes this shift in habitat use. This indicated that *M. natalensis* moved more into the non-preferred habitats (houses), even if the numbers caught inside houses were still relatively low.

Future studies should examine the potential impacts of including strategies targeting different pest species within integrated pest management plans. Such studies should explore the impacts on rodent abundance and rodent-induced damage, as well as the interplay between competitive pressures and resource availability. In the present study, differences between villages in the distribution of the two rodent species and their movement patterns could have influenced results; future studies should therefore consider using experimental designs that include pre- and post-treatment monitoring within villages. To ensure that consumption of RhB is not underreported, future studies should also endeavor to use a dark field room to examine animals for the presence of RhB. Here, we do not believe our approach of using hands to provide shade and enhance the visibility of fluorescence will have influenced our results, as the approach was used for both species and during all field trips.

In conclusion, our study highlights the importance of adopting an integrative and ecologically informed approach to rodent management in rural village settings, which accounts for species interactions and potential shifts in species distribution. While intensive in-house trapping proved effective in reducing *R. rattus* abundance, it also revealed habitat shifts by *M. natalensis*, which exploited more newly available resources within houses, as would be expected according to community ecology theory. These findings highlight the interconnectedness of rodent populations and the potential limitations of single-species or single-habitat control measures in addressing broader pest management challenges. It notably recognizes that control measures targeting one species can involuntarily alter habitat use by other species and encourage habitat encroachment by other species (Makundi et al. 1999). It also confirms that rodent species can exhibit flexible habitat use under variable conditions, adjusting movement patterns based on resource accessibility and competitive pressures (Massawe et al. 2011; Makundi and Massawe 2011). By tailoring rodent management to reflect the ecological and behavioral distinctions of different rodent species within communities, including their individual responses to management, we will be able to control pest rodent populations more successfully and efficiently, which will ultimately mitigate agricultural losses, reduce public health risks, and maintain ecological balance in rural communities
